# Supplementary material for: Lung microbiomes’ variable responses to dust exposure in mouse models of asthma
Source: mSphere. 2025 Oct 21;10(11):e00209-25. doi: 10.1128/msphere.00209-25 (PMC12645928; doi:10.1128/msphere.00209-25)
Supplement: Supplemental material — Graphical abstract and Fig. S1. [file msphere.00209-25-s0001.pdf]

Graphical Abstract

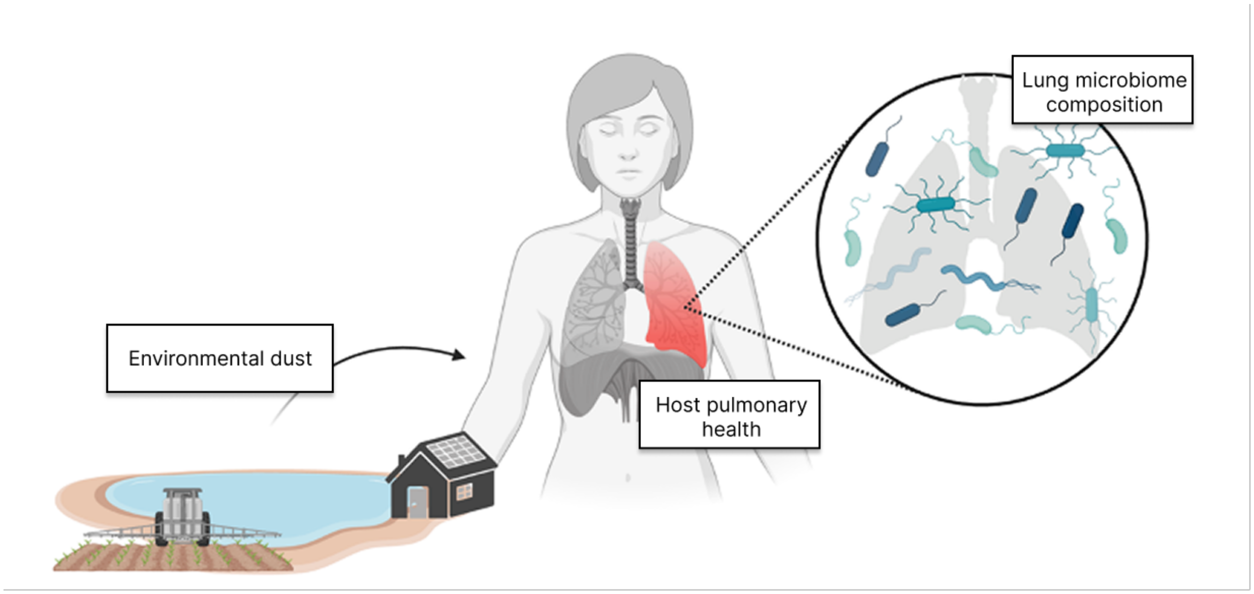

Supplementary Figure

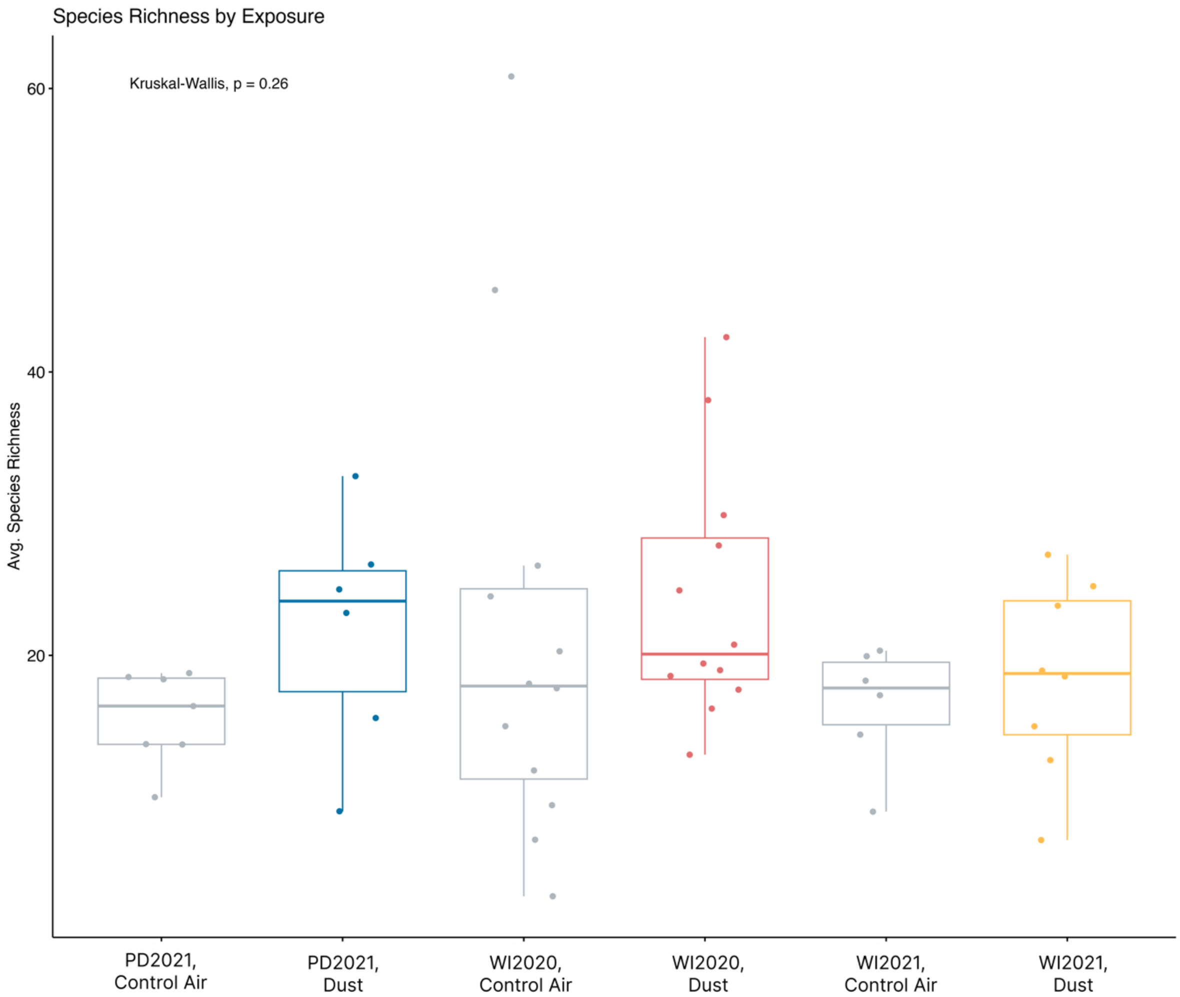

S1. Microbial taxa richness and dust exposure. Average taxa richness does not differ significantly among exposure groups (P=0.26).
